# Access, Use, and Patient-Reported Experiences of Emergency Care During the COVID-19 Pandemic: Population-Based Survey

**DOI:** 10.2196/30878

**Published:** 2021-09-08

**Authors:** Jodi Gray, Andrew Partington, Jonathan Karnon

**Affiliations:** 1 Flinders Health and Medical Research Institute College of Medicine and Public Health Flinders University Bedford Park Australia

**Keywords:** ED avoidance, emergency department avoidance, telehealth, COVID-19, access, usage, experience, patient-reported, emergency department, survey, telemedicine, barrier

## Abstract

**Background:**

An increase in the number of people presenting to emergency departments (EDs) is contributing to ED overcrowding. In the early stages of the COVID-19 pandemic, there was a significant reduction in the number of ED presentations in Australia, creating an opportunity to learn from patients’ experiences of alternative management options.

**Objective:**

The aim of this study is to report on the use and experience of health services by Australian adults experiencing a health issue during the COVID-19 pandemic for which they would have presented at an ED prior to the pandemic.

**Methods:**

An online survey was conducted in May 2020. Reported health issues were categorized using an existing classification system. Data collected included demographics, care pathways, levels of concern at times of health issue and survey completion, and patient-reported experiences with care.

**Results:**

A total of 1289 eligible respondents completed the survey. Almost 25% (309/1289) of respondents avoided an ED presentation, of which 58% (179/309) used an alternative form of health care and 42% (130/309) self-managed. Respondents making face-to-face or telehealth appointments with their general practitioner (GP) reported high levels of ED avoidance (135/286, 47%) and mostly positive experiences of care provided by GPs. A high proportion of those who self-managed reported high levels of concern at the time of completing the survey (42/130, 32%).

**Conclusions:**

Telehealth consultations with GPs may be a more promotable alternative to the ED beyond the COVID-19 pandemic, providing easier access to a doctor with access to patients’ medical histories than an appointment for a face-to-face consultation. GP telehealth consultations may also address barriers to accessing health care for those with potentially the greatest need. The reported use and positive experiences with GP telehealth appointments should inform further research on their appropriateness as an alternative to the ED.

## Introduction

There are increasing numbers of presentations to Australian public hospital emergency departments (EDs); in 2018/2019, there was a 4.2% increase in annual ED presentations to 8.4 million [1]. Morley et al [2] report increases in low-acuity ED presentations as one of the main input-based drivers of ED crowding. In Australia, almost 90% of GP consultations are fully subsidized by the government [3], but there is no fee for Australian residents who present at the ED of public hospitals. This likely facilitates low-acuity ED presentations. A recent Australian study in which general practitioners (GPs) held a regular GP consultation with patients immediately after a decision to discharge to home from the ED concluded that 20% to 40% of all ED presentations could potentially be diverted to primary care [4].

In the early stages of the COVID-19 pandemic, there was a significant reduction in the number of ED presentations in Australia [5], at least in part due to perceived infection risk in the ED. As a result of the pandemic, the Australian government introduced funding for GP telehealth consultations, which accounted for over 20% of GP consultations in 2020 [3]. The Australasian College for Emergency Medicine notes that “[t]here are many lessons to be learned from the response to COVID-19,” including responses relating to the management of health events for which individuals would have attended an ED prior to the pandemic [6].

While some reductions in ED presentations during the pandemic may be attributable to reductions in acute events due to social distancing and lockdown orders, it is unknown how many patients requiring care have sought alternative, non-ED care, and how many have experienced inappropriate care or even harm by avoiding the ED [7]. Worldwide, many have reported on the reduction in ED presentations observed during the COVID-19 pandemic [7-10]; however we found no studies that specifically explored whether patients sought out other health care services as an alternative to the ED and their experiences with those services. There has been some reporting of concurrent changes in health service utilization. For example, in the United Kingdom, the reduction in ED attendance during the COVID-19 pandemic has occurred alongside an increase in the number of ambulance callouts with treatment at the scene rather than transport to hospital; an increase in the number of calls to the NHS telephone helpline (NHS 111) but with fewer callers referred to an ED; and a reduction in the number of GP appointments despite the use of telehealth services where possible [7,11].

Evaluations of patient [12] and practitioner [13] experiences with expanded telehealth during the COVID-19 pandemic (eg, in general practice, allied health, and specialist care) have been largely positive; however, none have specifically looked at telehealth for ED avoidance during this pandemic period.

We report findings from a survey—undertaken during the early stages of the COVID-19 pandemic—of Australian adults who reported experiencing a health issue for which they would previously have presented at an ED. The survey data describe respondent characteristics, health issues experienced, care pathways accessed, and respondents’ experiences with care received. The aim of the survey was to identify potential lessons from the response to the pandemic to inform further research to improve emergency care in Australia.

## Methods

### Overview

An online survey (Multimedia Appendix 1) was designed to collect information on health-seeking behavior through people’s use of services during the early stages of the COVID-19 pandemic in Australia. Potential participants were asked to complete the survey if they had experienced a health issue for which they considered attending a hospital ED within the last four weeks. An additional survey question sought confirmation that respondents would have attended an ED for this issue prior to the pandemic. This paper follows the CHERRIES (Checklist for Reporting Results of Internet E-Surveys) checklist for the reporting of online surveys [14].

### Survey Design and Implementation

The survey instrument was designed by the authors, and the separate components of the survey were developed in turn. Existing classification systems for health issues were reviewed [15], but a free-text response was selected to reduce respondent burden with respect to health literacy and response times, with the aim of categorizing responses for analysis.

A total of four broad care pathways were defined:

1. Attended the ED as their first option
2. Attended the ED after contacting another (non-ED) health care provider first
3. Only contacted another (non-ED) health care provider
4. Self-managed (ie, did not seek any form of health care)

Respondents who contacted another health care provider were asked for further details. Likert scales were used to assess levels of concern at the times of the health event and the survey, as well as general health.

The 10-item Generic Short Patient Experiences Questionnaire (GS-PEQ) [16] was used to assess respondents’ experiences with care provided. Given the need to keep online surveys short and concise, a short-form patient-reported experience measures questionnaire was required. The GS-PEQ is based on the validated and reliable Nordic Patient Experiences Questionnaire (NORPEQ) [17] and other validated instruments used within the Norwegian health system [16]. The GS-PEQ assesses patient experiences using 10 questions with Likert scale responses.

The full survey underwent two rounds of online piloting with colleagues at Flinders University, followed by online piloting on May 5, 2020, with 53 panel provider respondents.

The survey was implemented online (Qualtrics [18]) during May 2020. Survey respondents were recruited via an International Organization for Standardization–accredited panel provider from May 5-14, 2020 (Dynata [19]), which enabled the recruitment of a large nationally representative sample within a short time period. A weblink to the survey was sent to all individuals registered with the provider, with the estimated time to complete the survey, but no information on the survey content. The weblink displayed the participant information sheet. Following this, potential respondents were asked for consent to participate before completing the screening question (Multimedia Appendix 1, page 1). The panel provider rewards respondents for completing surveys based on a structured incentive scheme that accounts for survey characteristics such as length and complexity.

Further recruitment was conducted from May 7-28, 2020, using Twitter and paid advertisements on Facebook (Multimedia Appendix 2). This allowed us to increase the number of young (18 to 24 years) female respondents, making the respondent sample more reflective of the Australian population of ED attenders (Multimedia Appendix 3) [1]. No incentives for survey completion were offered to Twitter or Facebook respondents.

### Survey Analysis

Incomplete and inconsistent survey responses were excluded from the analysis. This included where the respondent gave no answer describing the health issue, indicated they attended for a normal hospital admission (ie, not an ED attendance), or said they attended only a non-ED provider but when asked which provider, they indicated they attended the ED.

Free-text descriptions of the health issue were categorized into Berendsen Russell et al’s 17 presenting problem categories [15]. Descriptions with insufficient detail and categories with small numbers were merged into an “other” category, leaving nine categories for reporting (cardiovascular, gastrointestinal, infection, injury, mental health, musculoskeletal, neurology, respiratory, and other).

Descriptive statistics were undertaken in R (version 3.3.3; R Foundation for Statistical Computing) [20] on the following: respondent characteristics, reported health conditions, and level of concern at the time of the reported event for different care choices; changes in level of concern between the time of the event and the time of completing the survey for different starting levels of concern and care choices; and respondent-reported experiences (GS-PEQ) for patients receiving face-to-face and telehealth GP appointments.

The distributions of responses for different care choices are presented (eg, the proportion of respondents in each age group category that presented to an ED first). Confidence intervals and *P* values are not presented to avoid the perception of p-hacking [21], as well as because the reported comparisons should be interpreted as descriptive and hypothesis generating, not as inferential and hypothesis testing.

### Ethics Approval and Funding

The project was approved by the Flinders University Social and Behavioural Research Ethics Committee (project number 8652).

This research was conducted by JG, AP, and JK at Flinders University for the National Health and Medical Research Council (NHMRC) Partnership Centre for Health System Sustainability (grant ID: 9100002) administered by the Australian Institute of Health Innovation, Macquarie University. Along with the NHMRC, the funding partners in this research collaboration are The Bupa Health Foundation; New South Wales Ministry of Health; Department of Health, Western Australia; and The University of Notre Dame Australia. Funders provided financial support for this research but did not have any input into the research project or manuscript production. The authors hold all data for the project.

## Results

### Survey Respondents

A total of 10,754 potential respondents viewed the information sheet for the survey; of these, 10,019 (93%) consented to participate, and 1920 (18%) consented and met the eligibility criteria (Table 1). Of the eligible respondents, 264 were excluded as their surveys were incomplete or inconsistent and 367 were excluded as the respondent stated that they would not have chosen to attend the ED for the stated health issue prior to the COVID-19 pandemic. Of the resulting final 1289 (12%) respondents, most were recruited through the panel provider (1104/1289, 86%; Multimedia Appendix 3).

**Figure 1:**
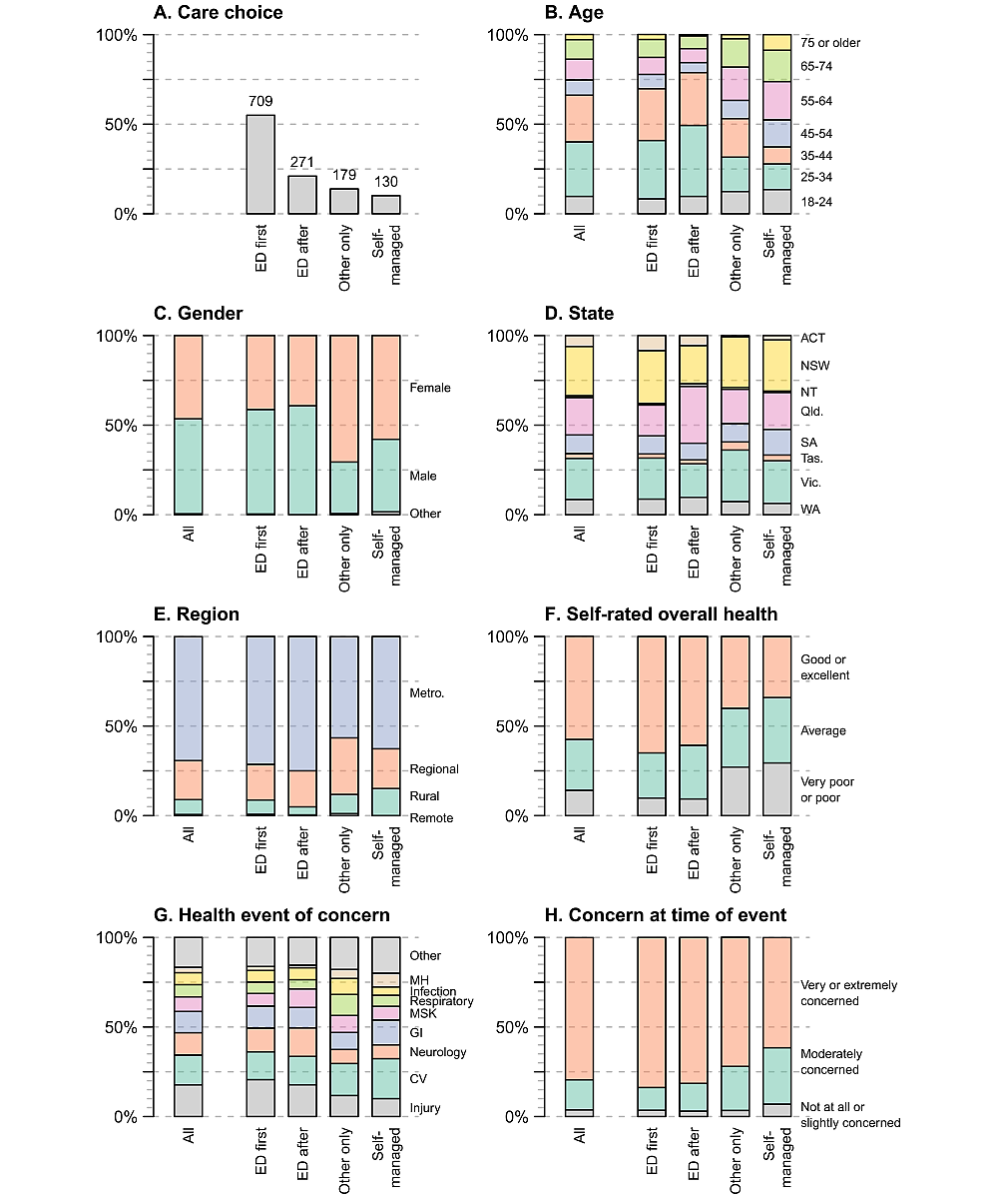
Respondent characteristics. Characteristics of all respondents included in the analysis and for respondents by care choice. Panels show data by (A) care choice, (B) age, (C) gender, (D) state, (E) region, (F) self-rated overall health, (G) health event of concern, and (H) concern at time of event. ACT: Australian Capital Territory; CV: cardiovascular; ED: emergency department; GI: gastrointestinal; MH: mental health; MSK: musculoskeletal; NSW: New South Wales; NT: Northern Territory; Qld.: Queensland; SA: South Australia; Tas.: Tasmania; Vic.: Victoria; WA: Western Australia.

Figure 1 displays the characteristics of all respondents and respondents by care choice. The eligible survey respondents were representative of the gender and geographical location of Australians who presented at an ED in 2018-2019, though the age distribution was different [1]. Primarily, the survey included smaller proportions of respondents aged 75 years and older and aged between 18 and 24 years, and a larger proportion of those aged between 25 and 44 years (Multimedia Appendix 3). A total of 15 respondents were missing demographic characteristic data and are excluded from percentage calculations.

**Table 1:** Respondent recruitment.

Recruitment steps	Panel provider, n (%)	Facebook and Twitter, n (%)	Total, n (%)
Viewed information sheet	10,386 (100)	368 (100)	10,754 (100)
Did not consent to participate^a^	730 (7.0)	5 (1.4)	735 (6.8)
Did not meet screening criteria^a^	8086 (77.9)	13 (3.5)	8099 (75.3)
Eligible (ie, consented and met screening criteria^a^)	1570 (15.1)	350 (95.1)	1920 (17.9)
Excluded from analysis^b,c^	466 (29.7)	165 (47.1)	631 (32.9)
Included in analysis^c^	1104 (70.3)	185 (52.9)	1289 (67.1)

^a^Percentage calculated using the number who viewed the information sheet as the denominator.

^b^Responses were excluded from the analysis if the survey answers were incomplete or inconsistent, or the respondent would not have chosen to attend the ED for the stated health issue prior to the COVID-19 pandemic. Examples of incomplete and inconsistent survey responses include when the respondent gave no answer describing the health issue, indicated they attended for a normal hospital admission (ie, not an ED attendance), or indicated they attended only a non-ED provider but when asked which provider, they indicated they attended the ED).

^c^Percentage calculated using the number eligible as the denominator.

Respondents resided in all states and territories of Australia and across metropolitan (882/1274, 69%) and nonmetropolitan areas (392/1274, 31%). More than half of respondents rated their health as good or excellent (733/1274, 58%). The most common health events reported by respondents were categorized as an injury (228/1274, 18%), cardiovascular conditions (214/1274, 17%), neurological conditions (161/1274, 13%), or gastrointestinal conditions (153/1274, 12%; see Multimedia Appendix 4 for further details and health event subcategories).

### Care Choices

**Figure 2:**
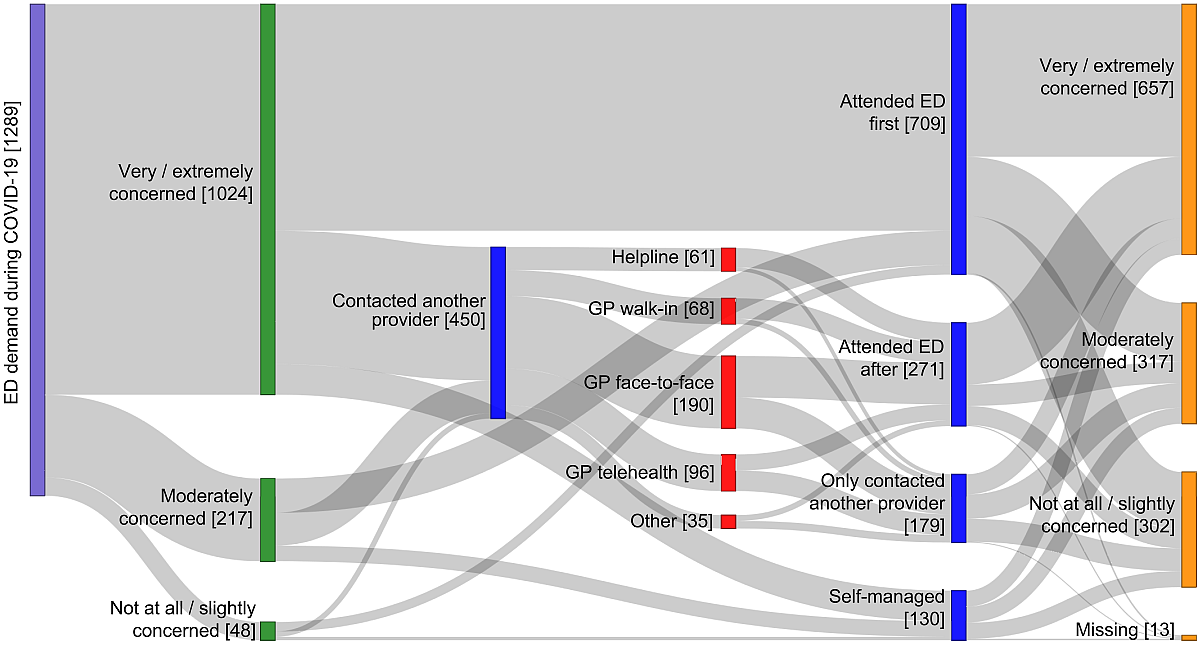
Respondents' levels of concern and care choices. Sankey figure mapping respondents' level of concern at the time of the health event (green bars), their care choices (blue and red bars), and level of concern at the time of completing the survey (orange bars). An interactive version of this figure is available in Multimedia Appendix 5. ED: emergency department; GP: general practitioner.

More than half of the respondents attended an ED as their first option (709/1289, 55%; Figure 1A; Figure 2; interactive version of Figure 2 in Multimedia Appendix 5). The remaining respondents delayed or completely avoided the ED: 21% (271/1289) reported attending an ED after contacting another health care provider, 14% (179/1289) only contacted another health care provider and did not attend an ED, and 10% (130/1289) self-managed their condition without contacting any provider.

A range of health care providers (Figure 2; Multimedia Appendix 5) were contacted by the respondents who either delayed attending the ED (contacted another provider first) or avoided attending the ED (only contacted another provider). Health care services used by these respondents were predominantly face-to-face GP appointments (190/450, 42%), telehealth appointments with GPs (96/450, 21%), attendance at GP walk-in clinics (68/450, 15%), and phoning a helpline (61/450, 14%). Other providers respondents contacted (35/450, 8%) included specialists (eg, their nephrologist, cardiologist, psychiatrist), allied health providers (eg, physiotherapist, dentist), pharmacists, or an ambulance service.

Of the respondents who contacted a helpline, 84% (51/61) went on to attend an ED, as did 79% (54/68) of respondents who attended a GP walk-in clinic. Fewer of the respondents who attended a face-to-face appointment with a GP (109/190, 57%) or a telehealth appointment with a GP (42/96, 44%) went on to attend the ED.

### Care Choices and Demographics

A younger cohort either attended the ED first or attended the ED after seeing another provider (72% of ED attenders were aged under 45 years, 701/971 [9 missing]), while 41% of those who avoided the ED or self-managed their health condition were aged 55 years or over (125/303 [6 missing]). A higher proportion of male respondents (410/675, 61%) compared to female respondents (290/593, 49%) reported attending the ED first. With some exceptions, the use of different health care types was fairly consistent across all states and geographic areas. Self-rated overall health (not related to the health issue of concern) was reported as good or excellent by more of those who attended the ED first (456/703 [6 missing], 65%) or attended the ED after contacting another provider (163/268 [3 missing], 61%), compared to those who avoided ED attendance (contacted another provider: 71/177 [2 missing], 40%; self-managed: 43/126 [4 missing], 34%).

There was some variation in care choices across health conditions. Injury was the most frequently reported health condition for those attending the ED (ED first: 146/709, 21%; ED after contacting another provider: 48/271, 18%), while cardiovascular conditions were most frequently reported for those only contacting another provider (32/179, 18%) or self-managing (29/130, 22%).

### Care Choices and Level of Concern

The majority of respondents (1024/1289, 79%) reported being very or extremely concerned at the time of the health event (Figure 1H; Figure 2; Multimedia Appendix 5; Multimedia Appendix 6). Of those who attended the ED first, 84% (594/709) were very or extremely concerned at the time of the event, compared to 82% (221/271) of those who attended the ED after contacting another health care provider, 72% (129/179) of those who contacted another provider only, and 62% (80/130) of those who self-managed.

A large number of respondents (607/1276 [13 missing], 48%) started and remained “very or extremely concerned” about the health issue (Multimedia Appendix 6). This sustained high level of concern was observed most prominently among those who either attended the ED as their first choice (373/705 [4 missing], 53% of this group) or went to the ED after having contacted another provider (152/268 [3 missing], 57%), compared to 27% (47/177 [2 missing]) of those who only contacted another provider and 28% (35/126 [4 missing]) of those who self-managed. Increased levels of concern were reported by 5% (58/1276 [13 missing]) of respondents overall, with the proportion highest for those who self-managed their health condition (8/126 [4 missing], 6%).

### Care Choices and Patient-Reported Experiences

From the GS-PEQ, overall satisfaction with the non-ED health care service was similar for respondents who went on to attend the ED (154/271 satisfied, 57%) and those who only contacted another provider (106/179 satisfied, 59%), as were the levels of overall dissatisfaction (later attended ED: 33/271 dissatisfied, 12%; another provider only: 25/179 dissatisfied, 14%).

**Figure 3:**
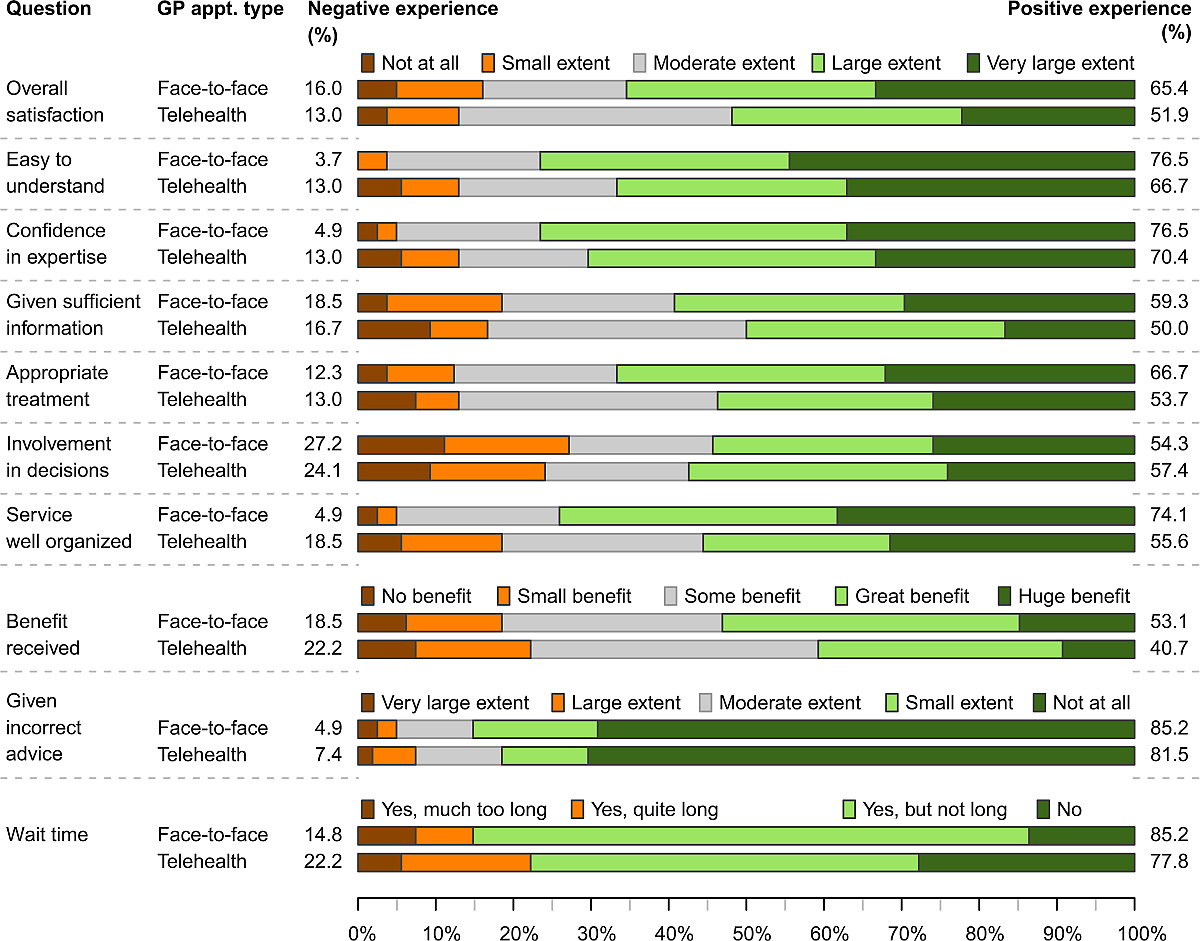
Patient experiences with care for those who only contacted another provider (GP face-to-face and GP telehealth appointments). The 10-item Generic Short Patient Experiences Questionnaire uses Likert scales to assess patient experiences with the non-ED care received. Negative experience (dissatisfied) includes the two most negative responses. Positive experience (satisfied) includes the two most positive responses. The wait time question used a 4-point Likert scale, while all other questions used a 5-point Likert scale. ED: emergency department; GP: general practitioner.

For those who only contacted another health care provider, Figure 3 reports patients’ experiences with care for those who accessed a GP via a telehealth appointment (54/179) and those who attended a face-to-face appointment with a GP (81/179). Examining overall satisfaction, telehealth respondents reported being satisfied (28/54, 52%) at a lower frequency than participants attending a face-to-face appointment (53/81, 65%). For almost all components of care, a larger proportion of the respondents attending face-to-face appointments reported positive experiences with the care provided compared to telehealth respondents. Negative experiences were more frequently reported for telehealth compared to face-to-face for ease of understanding (telehealth: 7/54, 13%; face-to-face: 3/81, 4%), confidence in the provider’s expertise (telehealth: 7/54, 13%; face-to-face: 4/81, 5%), whether the health service was well organized (telehealth: 10/54, 19%; face-to-face: 4/81, 5%), and waiting times (telehealth: 12/54, 22%; face-to-face: 12/81, 15%).

### Respondents Who Self-managed

The 10% of respondents (130/1289) who reported that they did not seek any form of health care may be of particular concern. These are respondents who would previously have presented at an ED, but due to the COVID-19 pandemic, they self-managed their health issue. Compared to respondents reporting alternative care choices, these respondents were more than twice as likely to be older than 65 years of age (self-managed 33/126 [4 missing], 26% versus sought any care 143/1148 [11 missing], 12%), report very poor or poor general health (37/126 [4 missing], 29% versus 142/1147 [11 missing], 12%), and live in a rural area/small town (19/126 [4 missing], 15% versus 87/1148 [11 missing], 7.6%).

Respondents not seeking care were also more likely to be female (73/126 [4 missing], 58% versus 520/1148 [11 missing], 45%), and to report mental health (10/130, 7.7% versus 29/1159, 2.5%) or cardiovascular (29/130, 22% versus 185/1159, 16%) conditions of concern.

Over 60% of self-managed respondents (80/130) reported being very or extremely concerned at the time of the reported health event, with 33% (42/126 [4 missing]) reporting being very or extremely concerned about their stated health issue at the time of survey completion, and an additional 33% (42/126 [4 missing]) reporting being moderately concerned at the time of survey completion.

## Discussion

### Principal Findings

This paper has reported findings from a large survey undertaken in May 2020 of 1289 adult Australians who reported experiencing a health issue in the last four weeks, for which they would have attended a hospital ED prior to the COVID-19 pandemic.

Overall, 35% (450/1289) of respondents contacted another health care provider, of whom 60% (271/450) went on to present at an ED. This means 14% of all respondents (179/1289) sought health care from an alternative source to the ED and avoided presenting at an ED. The avoidance of an ED presentation to the date at which the survey was completed, combined with low levels of dissatisfaction with the health care received suggests around 1 in 7 patients with a perceived need for emergency care can be cared for satisfactorily outside of an ED. 

Among survey respondents who contacted another provider, 79% contacted a GP (354/450), with an ED presentation avoided by 43% and 56% of respondents who contacted a GP for a face-to-face (81/190) or telehealth (54/96) consultation, respectively. The avoidance of an ED presentation by around half of all patients making a booked GP consultation implies more scope to promote the use of GP consultations as an alternative to presenting at an ED. However, the effectiveness of campaigns to promote use of primary care as an alternative to EDs may be limited by accessibility barriers and funding incentives that may promote ED attendance [2,22]. The ongoing availability of GP consultations via telehealth may provide an effective and attractive alternative to ED presentations, especially if bulk billed. Bulk-billed GP telehealth consultations mean that patients do not need to travel to an ED department and experience long waiting times in the ED, while still providing a consultation with a medically trained doctor that is free at the point of care for patients. Face-to-face GP consultations require travel and that may tip the balance toward an ED presentation. Another alternative is a telephone helpline, such as Healthdirect [23], but this was rarely reported in survey responses, and of those who did use a helpline, 84% (51/61) subsequently presented to the ED. In comparison to a helpline service, GP telehealth consultations are with doctors who can provide definitive medical advice, and long waiting times on the phone are avoided because an appointment time is made.

While further research is required to assess the acceptability and appropriateness of GP telehealth consultations as an alternative to ED presentations, preliminary evidence suggests telehealth has become an acceptable and viable method of providing a broad range of health care services. A survey of Australian patients who accessed telehealth services during the COVID-19 pandemic found 62% reported their experience as “as good as” or “better than” face-to-face appointments, with many reporting that continuing telehealth services would be useful postpandemic [12]. Clinicians across general practice, allied health, and specialist services have described how changes to managerial and medical culture, combined with changes to funding of telehealth during the pandemic, have legitimized telehealth services, increasing confidence in and acceptance of this technology [13].

A finding of concern is that 10% of respondents (130/1289) did not seek any form of health care, with high reported rates of concern about their stated health issue at the time of survey completion. Self-management was more common in older individuals and those with poor or very poor general health—groups at heightened risk of COVID-19 severe illness. The introduction of government funding for GP telehealth consultations was designed to provide concerned patients with a safe method of receiving health care during the pandemic, but the finding that 1 in 10 individuals with potentially urgent health care needs chose to self-manage their health condition suggests barriers to the use of telehealth should be further explored and addressed. Isautier et al [12] found 1.4% (19/1369) of their survey participants were unable to access telehealth services during the pandemic. Reasons included that their GP or health care professional did not provide telehealth services, appointments were not available when required, the patient did not have internet access, or the patient felt the process was too complicated.

### Limitations

The recruitment of survey respondents via an online survey resulted in the underrepresentation of persons in the youngest and oldest age categories: 3% (37/1274) and 10% (123/1274) of survey respondents were aged 75 years or older and 18 to 24 years, respectively, compared to 16.0% and 16.3% of people presenting at Australian EDs in 2018-2019, respectively (Multimedia Appendix 3) [1]. These differences should be taken into account when interpreting the survey findings; for example, the underrepresentation of older respondents may have underestimated the true proportion of people who avoided presenting at an ED, and in particular, those who did not seek health care. The benefits of using an online survey include the collection of data from a large sample (in this case, 1289 eligible respondents).

The nature of the survey data collected, in particular, the reliance on self-reported health conditions and the lack of a validated measure of urgency means that the application of inferential statistical analyses was not appropriate. Self-reported surveys are a valid source of data to describe the demographic characteristics of adults who experienced events for which they would have attended an ED prior to the COVID-19 pandemic and a general classification of the associated health problem. Self-report is also appropriate for describing patients’ experience of alternative forms of health care. We propose that the data are sufficient to inform hypotheses to be addressed by further research.

Building on the findings of the survey reported in this paper, further research might focus on defining, facilitating, and promoting the use of GP services for a range of conditions as an alternative to the ED. Such research might focus on musculoskeletal, respiratory, and cardiovascular conditions, which were most commonly reported by respondents who received health care while avoiding the ED. Facilitation options include incentives for bulk billing for “ED avoidance” consultations, while promotional activities might aim to improve health literacy using stories describing the experiences of people who avoided the ED during the COVID-19 pandemic, as well as the promotion of telehealth as a more convenient alternative to presenting at an ED.

The other key focus for further research that has been highlighted by the survey findings is the cohort of individuals who perceived a need for emergency care, but did not seek health care from any provider. Barriers and facilitators to accessing health care by this group should be investigated, with a particular focus on GP telehealth consultations, for which funding was introduced to facilitate better access. Such research can inform improved access to health care in times of public health emergencies as well as in “normal” times, as the pandemic is likely to have exacerbated an existing access issue [24].

### Conclusions

The reported survey of adult Australians who experienced a health issue for which an ED presentation would have been made prior to the COVID-19 pandemic provides insights into the effects of the COVID-19 pandemic on the demand and use of health care in Australia. The survey has provided evidence of positive experiences with alternatives to the ED, including telehealth consultations with GPs. It has also identified a cohort of generally older people with poorer general health for whom health system responses to support access to health care during the pandemic may have been insufficient. These findings provide a starting point for further research that should inform important policy responses that build on and respond to the effects of the COVID-19 pandemic on the health system.

## Appendix

Multimedia Appendix 1 Survey tool.

Multimedia Appendix 2 Survey advertisements used for social media.

Multimedia Appendix 3 Respondent characteristics for additional groups.

Multimedia Appendix 4 Detailed coding of problem description using Berendsen Russell’s categories and subcategories.

Multimedia Appendix 5 Interactive version of Figure 2. Respondents' levels of concern and care choices.

Multimedia Appendix 6 Sankey plots of level of concern at the time of the health event and level of concern at time of completing the survey by health care choice.

## Abbreviations

CHERRIES: Checklist for Reporting Results of Internet E-Surveys
ED: emergency department
GP: general practitioner
GS-PEQ: Generic Short Patient Experiences Questionnaire
NORPEQ: Nordic Patient Experiences Questionnaire
