# Supplementary material for: Access, Use, and Patient-Reported Experiences of Emergency Care During the COVID-19 Pandemic: Population-Based Survey
Source: JMIR Hum Factors. 2021 Sep 8;8(3):e30878. doi: 10.2196/30878 (PMC8428819; doi:10.2196/30878)
Supplement: Multimedia Appendix 1 [file humanfactors_v8i3e30878_app1.pdf]

Appendix A. Survey tool

[The information sheet was shown as the first page of the survey]

Research Project "What is the impact of the COVID-19 pandemic on emergency health care in Australia?" INFORMATION SHEET

Description of the study This a survey of people who experienced a health issue for which they considered going to a hospital emergency department over the last 4 weeks (during the COVID-19 pandemic).

Purpose of the study The survey data will describe how people managed health issues arising during the COVID-19 pandemic to inform potential improvements to the delivery of care after the COVID-19 pandemic.

What will I be asked to do? You are invited to complete a survey that includes questions about the health issue for which you considered going to a hospital emergency department and your level of concern about your health. It will also ask about contacts with health professionals, your experience with the health care provided and out-of-pocket expenses. The survey will take 5 to 10 minutes to complete.

What benefit will I gain from being involved in this study? The sharing of your experiences receiving health care during the COVID-19 pandemic will be used to identify areas in which the delivery of health care could be improved.

Will I be identifiable by being involved in this study? We do not need your name and you will be anonymous. Any identifying information will be removed, and your comments will not be linked directly to you. All information and results obtained in this study will be stored in a secure way, with access restricted to relevant researchers.

Are there any risks or discomforts if I am involved? The research team anticipate few risks from your involvement in this study. However, some participants could experience emotional discomfort. If you have any concerns regarding anticipated or actual risks or discomforts, please raise them with a member of the research team:

- Dr Clara Pham: clara.pham@flinders.edu.au
- Professor Jonathan Karnon: jonathan.karnon@flinders.edu.au

We, the researchers, do not endorse non-attendance at an ED, and would encourage anyone with a health concern to seek the appropriate medical advice/assistance.

How do I agree to participate? The first survey question asks if you consent to participate. You may refuse to answer any questions, and you are free to withdraw from the survey at any time without effect or consequences.

This research project has been approved by the Flinders University Social and Behavioural Research Ethics Committee in South Australia (Project number 8652). For queries regarding the ethics approval of this project please contact the Executive Officer of the Committee via telephone on +61 8 8201 3116 or email human.researchethics@flinders.edu.au

---

[Consent question]

I have read and I understand the Participant Information Sheet. (Please select one answer)

- ◯ I voluntarily consent to be a participant in this research (1)
- ◯ I do not consent to be a participant in this research (2)

Display This Question:
If I have read and I understand the Participant Information Sheet. (Please select one answer) = I voluntarily consent to be a participant in this research

[Primary screening question]

Have you experienced a health issue for which you considered going to a hospital Emergency Department in the last four weeks?

- ◯ Yes (1)
- ◯ No (2)

End of Block: Consent

Start of Block: Screen question

Please provide a short description of the health issue for which you considered going to a hospital Emergency Department (some examples might include: "chest pain", "I slipped on the tiles and fell and hit my head")

____

How long ago did you experience this health issue?

____

At the time, how concerned were you about the health issue?

- ◯ Extremely concerned (28)
- ◯ Very concerned (29)
- ◯ Moderately concerned (30)
- ◯ Slightly concerned (31)
- ◯ Not concerned (32)

Did you go to a hospital Emergency Department for this health issue?

- Yes, the Emergency Department was the first health care provider I contacted (1)
- Yes, but I contacted a different health care provider first (2)
- No, but I contacted a different health care provider (4)
- No, and I did not seek any form of health care for this health issue (5)

Display This Question:
If Did you go to a hospital Emergency Department for this health issue? = No, and I did not seek any form of health care for this health issue

[Used as a secondary screening question during analysis]

Do you think you would you have gone to a hospital Emergency Department if you had experienced this health issue before the COVID-19 pandemic?

- Yes (1)
- No (2)

Display This Question:
If Do you think you would you have gone to a hospital Emergency Department if you had experienced th... = Yes

Did you not go to a hospital Emergency Department because you were concerned about being infected with COVID-19?

- Yes (1)
- No (2)

Display This Question:
If Did you go to a hospital Emergency Department for this health issue? = No, but I contacted a different health care provider

[Used as a secondary screening question during analysis]

Do you think you would you have gone to a hospital Emergency Department instead of contacting a different health care provider if you had experienced this health issue before the COVID-19 pandemic?

- Yes (1)
- No (2)

Display This Question:
If Do you think you would you have gone to a hospital Emergency Department instead of contacting a d... = Yes

Did you not go to a hospital Emergency Department because you were concerned about being infected with COVID-19?

- Yes (1)
- No (2)

Display This Question:
If Did you go to a hospital Emergency Department for this health issue? = Yes, but I contacted a different health care provider first

[Used as a secondary screening question during analysis]

Do you think you would you have gone to a hospital Emergency Department before contacting a different health care provider if you had experienced this health issue before the COVID-19 pandemic?

- Yes (1)
- No (2)

Display This Question:
If Do you think you would you have gone to a hospital Emergency Department before contacting a diffe... = Yes

Did you not go to a hospital Emergency Department before seeing another health care provider because you were concerned about being infected with COVID-19?

- Yes (1)
- No (2)

End of Block: Screen question

Start of Block: Health care use 1

Display This Question:
If Did you go to a hospital Emergency Department for this health issue? = No, but I contacted a different health care provider

Which health care provider(s) did you contact?

- ▢ Rang a helpline, for example, Healthdirect (8)
- ▢ Made a face-to-face appointment with a GP (1)
- ▢ Attended a walk-in GP clinic (7)
- ▢ Spoke to a GP on the phone or using a computer (a telehealth consultation) (2)
- ▢ Visited a pharmacy (4)
- ▢ Rang a pharmacy (5)
- ▢ Other (6) ____

Display This Question:
If Did you go to a hospital Emergency Department for this health issue? = Yes, but I contacted a different health care provider first

Which health care provider(s) did you contact before going to a hospital Emergency Department?

- ○ Rang a helpline, for example, Healthdirect (1)
- ○ Made a face-to-face appointment with a GP (2)
- ○ Attended a walk-in GP clinic (3)
- ○ Spoke to a GP on the phone or using a computer (a telehealth consultation) (4)
- ○ Visited a pharmacy (5)
- ○ Rang a pharmacy (6)
- ○ Other (7) ____

End of Block: Health care use 1

Start of Block: Patient reported experience

Display This Question:
If Did you go to a hospital Emergency Department for this health issue? = Yes, the Emergency Department was the first health care provider I contacted

Thinking about the health care you received at the hospital Emergency Department.

Display This Question:
If Did you go to a hospital Emergency Department for this health issue? = Yes, but I contacted a different health care provider first

Thinking about the health care you received before going to a hospital Emergency Department.

Display This Question:
If Did you go to a hospital Emergency Department for this health issue? = No, but I contacted a different health care provider

Thinking about the health care you received.

Display This Question:
If Did you go to a hospital Emergency Department for this health issue? != No, and I did not seek any form of health care for this health issue

Did the health professional(s) that you contacted talk to you in a way that was easy to understand?

- ○ Not at all (1)
- ○ To a small extent (2)
- ○ To a moderate extent (3)
- ○ To a large extent (4)
- ○ To a very large extent (5)

Display This Question:
If Did you go to a hospital Emergency Department for this health issue? != No, and I did not seek any form of health care for this health issue

Did you have confidence in the health professional expertise?

- Not at all (1)
- To a small extent (2)
- To a moderate extent (3)
- To a large extent (4)
- To a very large extent (5)

Display This Question:
If Did you go to a hospital Emergency Department for this health issue? != No, and I did not seek any form of health care for this health issue

Did you get sufficient information about your health condition?

- Not at all (1)
- To a small extent (2)
- To a moderate extent (3)
- To a large extent (4)
- To a very large extent (5)

Display This Question:
If Did you go to a hospital Emergency Department for this health issue? != No, and I did not seek any form of health care for this health issue

At the time you received care, did you think the advice or treatment you received was appropriate?

- Not at all (1)
- To a small extent (2)
- To a moderate extent (3)
- To a large extent (4)
- To a very large extent (5)

Display This Question:
If Did you go to a hospital Emergency Department for this health issue? != No, and I did not seek any form of health care for this health issue

Were you involved in decisions regarding any treatment you received?

- Not at all (1)
- To a small extent (2)
- To a moderate extent (3)
- To a large extent (4)
- To a very large extent (5)

Display This Question:
If Did you go to a hospital Emergency Department for this health issue? != No, and I did not seek any form of health care for this health issue

Did you think the health service organisation(s) you contacted was well organised?

- Not at all (1)
- To a small extent (2)
- To a moderate extent (3)
- To a large extent (4)
- To a very large extent (5)

Display This Question:
If Did you go to a hospital Emergency Department for this health issue? != No, and I did not seek any form of health care for this health issue

Did you have to wait before you were able to talk to the health professional(s) you contacted?

- No (1)
- Yes, but not long (2)
- Yes, quite long (3)
- Yes, much too long (4)

Display This Question:
If Did you go to a hospital Emergency Department for this health issue? != No, and I did not seek any form of health care for this health issue

Overall, was the help and treatment you received satisfactory?

- ○ Not at all (1)
- ○ To a small extent (2)
- ○ To a moderate extent (3)
- ○ To a large extent (4)
- ○ To a very large extent (5)

Display This Question:
If Did you go to a hospital Emergency Department for this health issue? != No, and I did not seek any form of health care for this health issue

Overall, what benefit did you get from the care you received?

- ○ No benefit (1)
- ○ Small benefit (2)
- ○ Some benefit (3)
- ○ Great benefit (4)
- ○ Huge benefit (5)

Display This Question:
If Did you go to a hospital Emergency Department for this health issue? != No, and I did not seek any form of health care for this health issue

Looking back, do you believe that you were in any way given incorrect advice and treatment (according to your own judgement)?

- ○ Not at all (1)
- ○ To a small extent (2)
- ○ To a moderate extent (3)
- ○ To a large extent (4)
- ○ To a very large extent (5)

End of Block: Patient reported experience

Start of Block: Costs

How many dollars did you spend on managing the health issue for which you considered going to a hospital Emergency Department? (some examples might include payments to health professionals, for treatments and for medical tests)

____

Display This Question:
If Did you go to a hospital Emergency Department for this health issue? = Yes, the Emergency Department was the first health care provider I contacted
Or Did you go to a hospital Emergency Department for this health issue? = Yes, but I contacted a different health care provider first

When you went to a hospital Emergency Department, did you spend a night in a hospital bed?

- ◯ Yes (23)
- ◯ No (24)

Today, how concerned are you about the health issue for which you considered going to a hospital Emergency Department?

- ◯ Extremely concerned (1)
- ◯ Very concerned (2)
- ◯ Moderately concerned (3)
- ◯ Slightly concerned (4)
- ◯ Not concerned (5)

End of Block: Costs

Start of Block: Demographics

Generally, how would you rate your health?

- ◯ Excellent (11)
- ◯ Good (12)
- ◯ Average (13)
- ◯ Poor (14)
- ◯ Very poor (15)

What is your age category?

- 18 - 24 (2)
- 25 - 34 (3)
- 35 - 44 (4)
- 45 - 54 (5)
- 55 - 64 (6)
- 65 - 74 (7)
- 75 - 84 (8)
- 85 or older (9)

What is your gender?

- Male (1)
- Female (2)
- Other (3)

What State do you live in?

- Tasmania (1)
- Western Australia (2)
- New South Wales (3)
- Victoria (4)
- South Australia (5)
- Northern Territory (6)
- Australian Capital Territory (7)
- Queensland (8)

What type of area do you live in?

- ○ Metropolitan area / Large city (1)
- ○ Regional area / Small city or Large Town (2)
- ○ Rural area / Small Town (3)
- ○ Remote community (4)

End of Block: Demographics
