# Supplementary material for: Access, Use, and Patient-Reported Experiences of Emergency Care During the COVID-19 Pandemic: Population-Based Survey
Source: JMIR Hum Factors. 2021 Sep 8;8(3):e30878. doi: 10.2196/30878 (PMC8428819; doi:10.2196/30878)

Appendix 2: Survey advertisements used for social media

Facebook advertisement

Considered going to ED recently?

If you answered YES, please complete a survey designed by health researchers at Flinders University, which aims to inform improvements in the delivery of health care in the hospital and the community.

The online survey takes 5 to 7-minute to complete and asks you about what health care you used and your experience and satisfaction with the health care you received.

Please click on the following link to complete the survey

<https://qualtrics.flinders.edu.au/jfe/form/SV_cIvr60mkyn48EUl>

Insert image below for Facebook and twitter

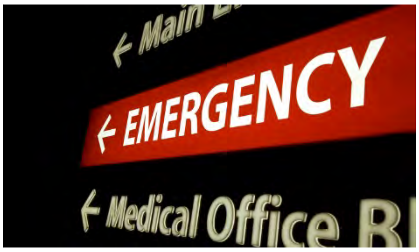

www1.racgp.org.au

Twitter advertisement

Flinders University @Flinders · 13 May 2020

Have you experienced a health issue for which you considered going to a hospital Emergency Department in the last four weeks? If yes, help us understand the patient perspective when seeking health care by completing this 5 to 7 minute survey. Click here: bit.ly/3cnlAr7

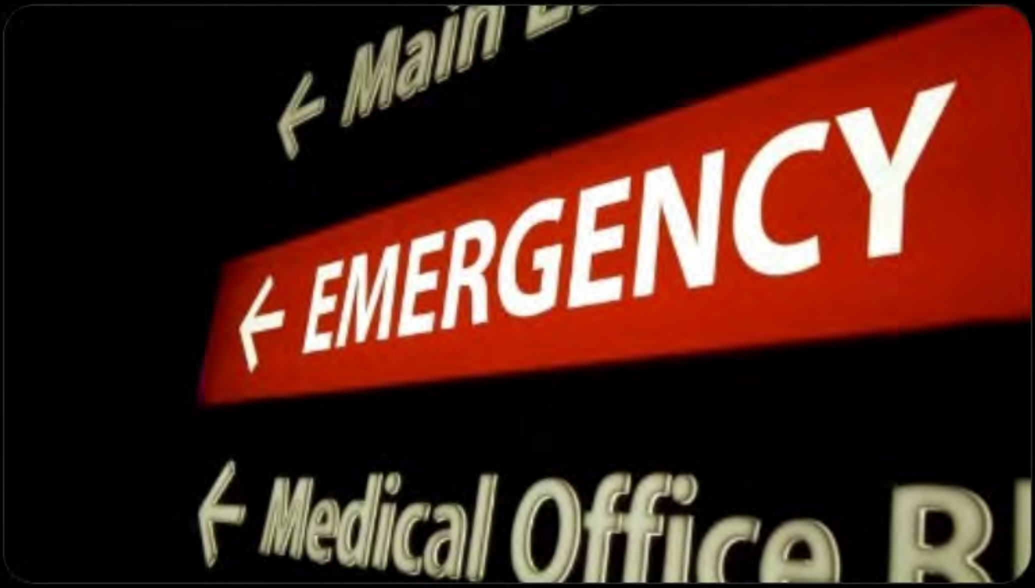

Supplement: Multimedia Appendix 2 [file humanfactors_v8i3e30878_app2.pdf]
