# Supplementary figures and images for: Access, Use, and Patient-Reported Experiences of Emergency Care During the COVID-19 Pandemic: Population-Based Survey
Source: JMIR Hum Factors. 2021 Sep 8;8(3):e30878. doi: 10.2196/30878 (PMC8428819; doi:10.2196/30878)

### Appendix 3. Respondent characteristics for additional groups

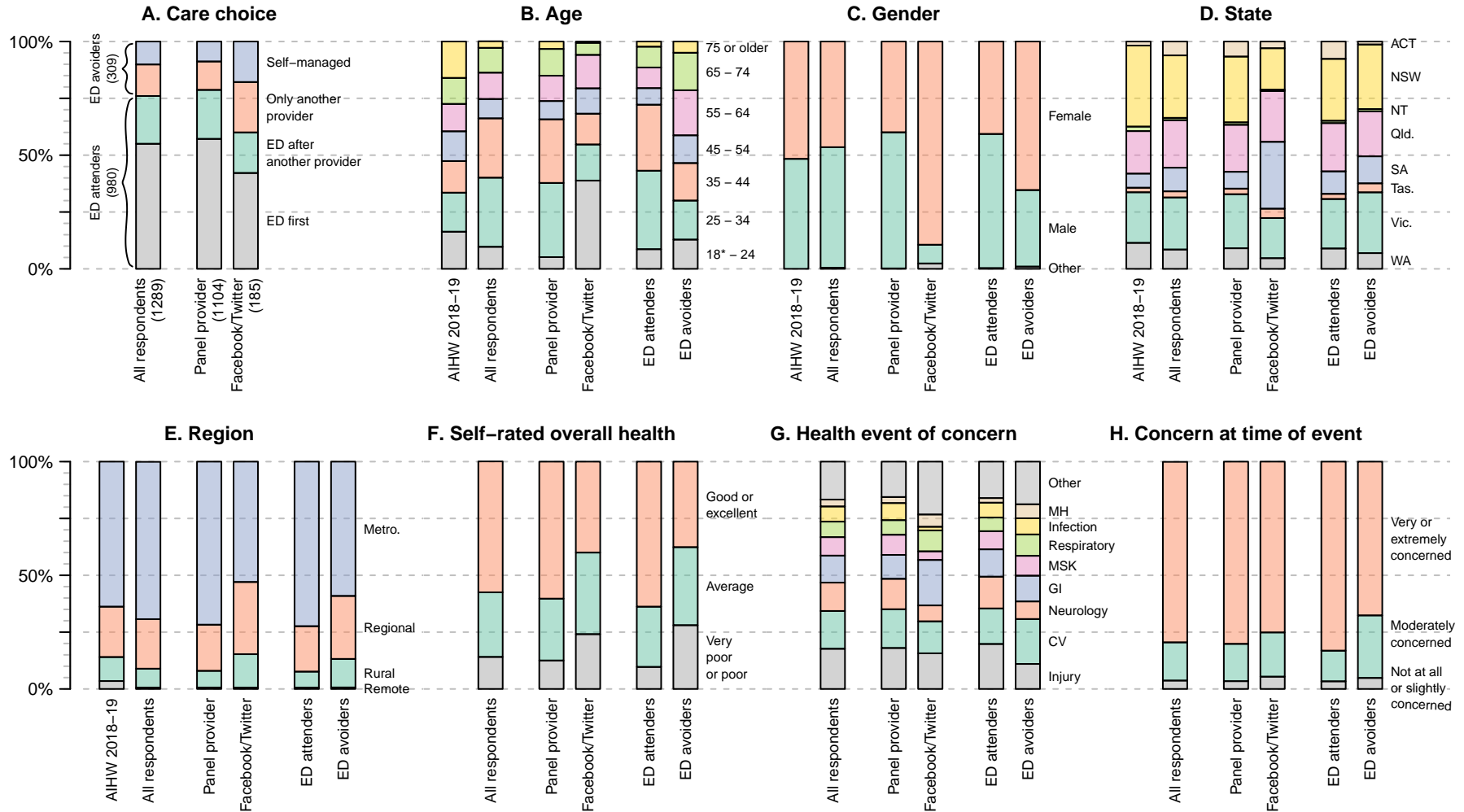

Supplement: Multimedia Appendix 3 [file humanfactors_v8i3e30878_app3.pdf]
