# Supplementary material for: Access, Use, and Patient-Reported Experiences of Emergency Care During the COVID-19 Pandemic: Population-Based Survey
Source: JMIR Hum Factors. 2021 Sep 8;8(3):e30878. doi: 10.2196/30878 (PMC8428819; doi:10.2196/30878)
Supplement: Multimedia Appendix 4 [file humanfactors_v8i3e30878_app4.pdf]

Appendix 4. Detailed coding of problem description using Berendsen Russell's categories and sub-categories

Categories	Sub-categories (coded based on patient description)	Count	Major category total
Cardiovascular	Arrhythmias, Palpitations	22	214
	Cardiac arrest	3	
	Chest pain	156	
	Collapse, (pre)syncope	7	
	Dizziness	12	
	Hypotension / Hypertension	6	
	Cardiovascular (NFD)	8	
Gastrointestinal	Abdominal pain	101	153
	Diarrhoea	5	
	Epigastric pain	1	
	Flank pain	9	
	Foreign body gastrointestinal tract	2	
	Nausea and or vomiting	6	
	PRB/maleana	4	
	Gastrointestinal (NFD)	25	
Infection	Cold and flu symptoms	48	86
	Fever	10	
	Fever, cough	6	
	Infection	22	
Injury	Alleged assault	2	228
	Alleged assault, sexual assault	1	
	Fall	84	
	MVA	4	
	Injury, bite	4	
	Injury, burn	10	
	Injury, compression	3	
	Injury, dislocation	5	
	Injury, fracture	38	
	Injury, haemotoma	3	
	Injury, hit / bump	20	
	Injury, impaled	1	
	Injury, laceration	30	
	Injury, penetrating	1	
	Injury, scratch	2	
	Injury, sprain	8	
	Injury, tear	1	
	Injury, by body location	10	
	Injury, other	1	
Mental health	Anxiety	9	39
	Depression	8	
	Self harm	1	
	Suicidal ideation	8	
	Mental health problem (NFD)	13	
Musculoskeletal	Musculoskeletal pain, lower limb	35	105
	Musculoskeletal pain, groin	1	
	Musculoskeletal pain, back	36	
	Musculoskeletal pain, neck	4	
	Musculoskeletal pain, ribs	1	

	Musculoskeletal pain, upper limb	14	
	Musculoskeletal pain, joints	9	
	Musculoskeletal pain (NFD)	4	
	Musculoskeletal (NFD)	1	
Neurology	Weakness	2	161
	Cerebrovascular accident (CVA)	5	
	Headache	141	
	Seizure	6	
	Neurological pain	3	
	Neurology (NFD)	4	
Respiratory	Asthma wheeze	29	88
	Choking/Foreign body respiratory tract	3	
	Cough, haemoptysis	5	
	Pleuritic pain	2	
	Shortness of breath	45	
	Respiratory (NFD)	4	
Other	Endocrine: Blood sugar issue	1	215
	Endocrine (NFD)	2	
	ENT, face: Dental problems	20	
	ENT, face: Ear problem	8	
	ENT, face: Eye problems	12	
	ENT, face: Mouth problem	2	
	ENT, face: Nose problem	3	
	Obstetrics and Gynaecology: Gynae issue	10	
	Obstetrics and Gynaecology: Per vaginal bleeding	1	
	Obstetrics and Gynaecology: Pregnancy related issues	10	
	Skin: Allergy	3	
	Skin: Bite/sting	2	
	Skin: Cellulitis	4	
	Skin: Mass/abscess	10	
	Skin: Rash	1	
	Skin: Swelling	1	
	Skin (NFD)	6	
	Toxicology: Alcohol related	1	
	Toxicology: Drug related	1	
	Toxicology: Overdose	1	
	Toxicology: Requesting detox	1	
	Toxicology (NFD)	1	
	Urology: Testicular problem	2	
	Urology: Urinary problem	12	
	Unwell: Unwell	16	
	Unwell: Other	68	
	Administration: Catheter related problems	1	
	Administration: Requests investigation	5	
	Administration: Requests medication, script or certificate	3	
	Administration: Review	7	
Total		1289	1289

NFD: no further details

Reference:

Berendsen Russell, S., M.M. Dinh, and N. Bell, Triage, damned triage… and statistics: Sorting out redundancy and duplication within an Emergency Department Presenting Problem Code Set to enhance research capacity. Australas Emerg Nurs J, 2017. 20(1): p. 48-52.
