# Supplementary material for: Access, Use, and Patient-Reported Experiences of Emergency Care During the COVID-19 Pandemic: Population-Based Survey
Source: JMIR Hum Factors. 2021 Sep 8;8(3):e30878. doi: 10.2196/30878 (PMC8428819; doi:10.2196/30878)
Supplement: Multimedia Appendix 5 [file humanfactors_v8i3e30878_app5.zip › Appendix 5. Interactive Figure 2.html]

Figure 2 (Interactive)


# Figure 2 [Interactive]. Respondents levels of concern and care choices

Sankey figure mapping respondents level of concern at the time of the health event [green], their care choices [blue and red] and level of concern at the time of completing the survey [orange].
