# Supplementary material for: Access, Use, and Patient-Reported Experiences of Emergency Care During the COVID-19 Pandemic: Population-Based Survey
Source: JMIR Hum Factors. 2021 Sep 8;8(3):e30878. doi: 10.2196/30878 (PMC8428819; doi:10.2196/30878)

Appendix 6. Sankey plots of level of concern at the time of the health event (green nodes) and level of concern at time of completing the survey (yellow nodes) by choice of health care

A. Attended the ED first

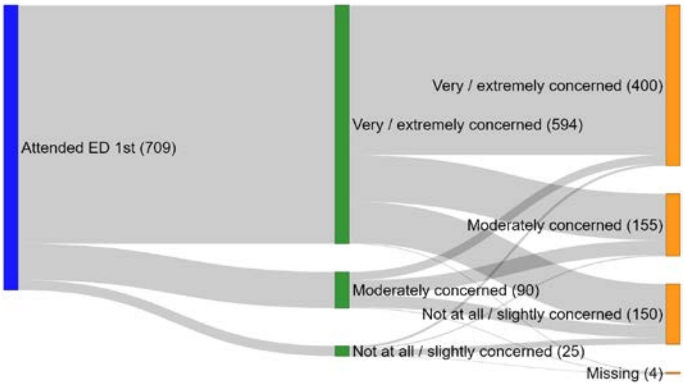

B. Attended the ED after contacting another provider

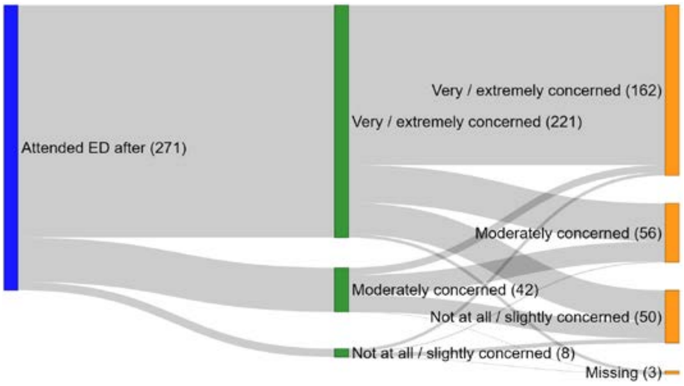

C. Only contacted another provider

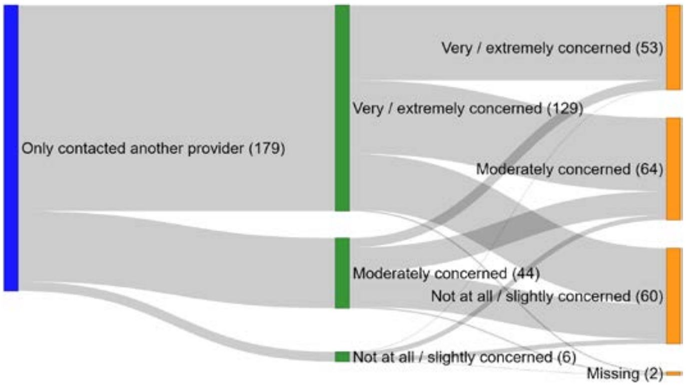

D. Self-managed

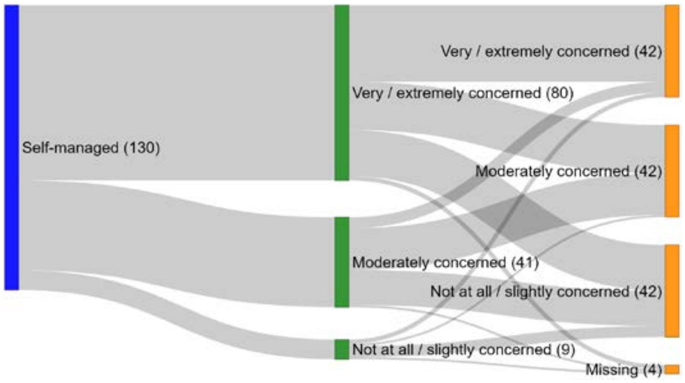

Supplement: Multimedia Appendix 6 [file humanfactors_v8i3e30878_app6.pdf]
